# Directed Evolution of Seneca Valley Virus in Tumorsphere and Monolayer Cell Cultures of a Small-Cell Lung Cancer Model

**DOI:** 10.3390/cancers15092541

**Published:** 2023-04-28

**Authors:** Shakeel Waqqar, Kai Lee, Blair Lawley, Timothy Bilton, Miguel E. Quiñones-Mateu, Mihnea Bostina, Laura N. Burga

**Affiliations:** 1 Department of Microbiology and Immunology, University of Otago, Dunedin 9016, New Zealandblair.lawley@pacificedge.co.nz (B.L.);; 2 Invermay Agricultural Centre, AgResearch, Mosgiel 9092, New Zealand

**Keywords:** Seneca Valley virus (SVV), directed evolution, viral quasispecies, cell culture, tumorsphere, cell monolayer, virus passage, deep sequencing, single nucleotide variants

## Abstract

**Simple Summary:**

Serial passaging of oncolytic viruses in a new host system can increase their infectivity and anti-cancer efficacy by the directed evolution of naturally occurring variants. RNA viruses have a high nucleotide substitution and proof-reading error rate due to the low fidelity of viral RNA-dependent RNA polymerase. The directed evolution of oncolytic RNA viruses is a practical strategy to select for virus variants with an increased therapeutic efficacy. The Seneca Valley virus (SVV) is a promising oncolytic virotherapy candidate for a range of human cancers. Here, we used deep genome sequencing to analyse viral genome changes during the serial passaging of the SVV in two cell culture models of small-cell lung cancer, i.e., monolayer cells and tumorsphere. We observed the improved infectivity of the SVV in tumorspheres over time associated with the accumulation of several mutations across the genome, possibly involved in the optimization of infectiousness in tumors.

**Abstract:**

The Seneca Valley virus (SVV) is an oncolytic virus from the picornavirus family, characterized by a 7.3-kilobase RNA genome encoding for all the structural and functional viral proteins. Directed evolution by serial passaging has been employed for oncolytic virus adaptation to increase the killing efficacy towards certain types of tumors. We propagated the SVV in a small-cell lung cancer model under two culture conditions: conventional cell monolayer and tumorspheres, with the latter resembling more closely the cellular structure of the tumor of origin. We observed an increase of the virus-killing efficacy after ten passages in the tumorspheres. Deep sequencing analyses showed genomic changes in two SVV populations comprising 150 single nucleotides variants and 72 amino acid substitutions. Major differences observed in the tumorsphere-passaged virus population, compared to the cell monolayer, were identified in the conserved structural protein VP2 and in the highly variable P2 region, suggesting that the increase in the ability of the SVV to kill cells over time in the tumorspheres is acquired by capsid conservation and positively selecting mutations to counter the host innate immune responses.

## 1. Introduction

The association of viruses with tumors has long been known, both as a causative agent and as a therapeutic option [1,2,3,4]. Viruses have evolved with their hosts and developed ingenious approaches to propagate and fight the immune responses [5,6]. By serial passaging within a host, viruses can acquire new properties such as increased infectivity [7,8]. This observation can be implemented for oncolytic viruses to improve therapeutic efficacy [6,7,9]. The role of genetic changes in altering the antitumor efficacy of oncolytic viruses has been investigated through genetic engineering and by the directed evolution of naturally occurring variants [10,11,12]. RNA viruses are known for their high substitution rates and errors in the proof-reading of newly generated viral genomes during an infection cycle due to the low fidelity of the viral RNA-dependent RNA polymerase (RdRp) [13,14]. As a result, the viral population consists of a mutant spectrum around the master genome known as viral quasispecies [15]. These errors are known to confer novel properties, which can help RNA viruses to adapt to diverse environments and can lead to improved viral stability and gain of fitness [16,17,18,19,20,21]. The directed evolution of oncolytic RNA viruses is a practical strategy to take advantage of natural selection to evolve viruses for increased therapeutic efficacy.

The Seneca Valley virus (SVV) has emerged as a promising oncolytic virotherapy candidate [22,23]. The SVV is a member of the *Picornaviridae* family, genus *Senecavirus*. The SVV has a single-stranded, positive-sense RNA genome consisting of 7280 nucleotides. The open reading frame is translated into a single polyprotein, which is further cleaved into 12 polypeptides in a L-4-3-4 fashion [24]. During the assembly process, three structural proteins VP0, VP1, and VP3 form pentamers that further self-assemble into the viral capsid, where VP0 is autocatalytically cleaved into VP2 and VP4. The SVV capsid has a typical picornavirus icosahedral structure with VP1, VP2, and VP3 forming the exterior and VP4 situated on the interior of the viral capsid [25,26]. The SVV oncolytic activity is mediated by the viral cellular receptor anthrax toxin receptor 1 (ANTXR1), also known as tumor endothelial marker 8 (TEM8) [27,28,29]. More than 60% of human solid cancer express ANTXR1/TEM8, which can be potentially targeted by SVV oncolytic virotherapy [27,30].

Three-dimensional (3D) cell culture models such as spheroids or tumorspheres are a better way to study the effects of therapeutics [31] and for studying the evolutionary trajectories of the viral quasispecies. These are cell formations growing in suspensions with or without the extracellular matrix, which can reproduce the architecture and metabolism of their original tissue to a certain extent. Furthermore, in comparison to 2D cell cultures (monolayers), tumorspheres have the advantage of reproducing the tumor microenvironment by causing hypoxia, nutrient gradient, apoptotic core, ATP distribution, and lactate accumulation [32,33,34,35,36,37].

Here, we report the first comparison of the evolution of the SVV in the small-cell lung cancer cell line (SCLC) H446 wild-type under tumorsphere or monolayer culture conditions. We have noticed the improved infectivity of the SVV in the tumorspheres over time and the accumulation of several mutations across the viral genome, possibly involved in the optimization of infectiousness in tumors.

## 2. Materials and Methods

*Cell culture and viral stock*. Small-cell lung cancer cell line H446 wild-type (H446wt) was propagated in Roswell Park Memorial Institute 1640 (RPMI1640) medium (Gibco, Billings, MT, USA), supplemented with 10% fetal bovine serum and incubated at 37 °C in 5% CO_2_. For virus propagation, H446wt cell culture was expanded into twelve T175 flasks. When reaching ~90% confluency, the cells were infected with SVV at a multiplicity of infection (MOI) of 1 and further incubated until complete cytopathic effect (CPE) was achieved. The cells were then freeze–thawed three times, followed by centrifugation at 10,000× *g* for 30 min to remove cell debris. The virus was spun down by centrifugation for 90 min at 120,000× *g* (Beckman Coulter Optima XPN-80 ultracentrifuge), and virus-containing pellets were resuspended overnight in a small volume of phosphate-buffered saline (PBS). The virus was separated by CsCl (1.33 g/mL) density-gradient ultracentrifugation for 18 h at 120,000× *g*. The fractions containing the virus were buffer-exchanged and concentrated with 100 kDa Amicon filters. The virus was confirmed by Coomassie staining and quantified by plaque formation assays. Full capsids were visualized on copper grids stained with phosphotungstic acid (PTA) using a Philips CM100 transmission electron microscope. Virus stock was used for subsequent experiments.

*Plaque formation assays*. H446wt cells were cultured into a 12-well plate in 1 mL RPMI1640 supplemented with 10% FBS. At confluency, the medium was removed, and the cells were incubated with ten-fold serial dilutions of virus for 1 h, then the inoculum was removed, and the cells were layered with 1 mL RPMI1640 supplemented with 2% FBS and 1% agar (Lonza, Basel, Switzerland). Viral plaques were observed after 18 to 24 h and visualized with 5 mg/mL MTT 3-(4,5-dimethylthiazol-2-yl)-2,5-diphenyltetrazolium bromide (Sigma, Burlington, MA, USA).

*Tumorsphere culture*. Single-cell suspension of H446wt cells were cultured at a density of 2000 cells/well in a low-attachment 96-well plate (Greiner Bio-one, Kremsmünster, Austria) in 0.1 mL tumorsphere medium (DMEM-F12 supplemented with B27 (Gibco; cat. 17504044), human epidermal growth factor (Sigma, Burlington, MA, USA), human basic fibroblast growth factor (SinoBiological; Beijing, China), and insulin (Merck, Darmstadt, Germany), and incubated for five days at 37 °C in 5% CO_2_. The average number of tumorspheres per well was determined by counting the total number of tumorspheres in six wells. The average number of cells per well was determined by nuclear staining with 1% Hoechst 33342 (Thermo Fisher Scientific; Waltham, MA, USA). Images were recorded using Olympus BX51M-IR microscope and the nuclei were counted with ImageJ.

*Serial passages of SVV*. Five-day-old tumorspheres were infected with SVV at MOI of 1. At 48 h post infection, whole contents of four infected and four control wells were collected and frozen at −80 °C. To release the virus, the tumorspheres were passed through three freeze–thaw cycles, sonicated for 1 min, and centrifuged at 10,000× *g* for 20 min. For virus passaging in the monolayer culture, H446 cells in T75 were infected with the stock SVV at MOI of 1. After 48 h of infection, flasks were transferred at −80 °C overnight, followed by four cycles of freeze–thawing at room temperature. The flask contents were spun at 10,000× *g* for 20 min to separate the cell debris. The viral titer in the supernatant was determined by plaque formation assays and the virus concentration was adjusted to MOI of 1 for the following cycle of infection. Clear supernatant from both tumorsphere and monolayer passages was assessed for virus concentration by plaque assays and the next cycle of infection was set at an MOI of 1.

*Cell viability assay*. For each viral passage, four wells of each infected and control tumorspheres were treated with 20 µL of 5 mg/mL MTT and incubated at 37 °C in 5% CO_2_ followed by addition of 10% SDS and 0.01 M HCl. The absorbance was recorded using a Bio-Rad microplate reader.

*Deep (next-generation) sequencing (NGS)*. SVV samples collected from each monolayer (SVVml) and tumorsphere (SVVts) passaging were used for RNA extraction using QIAmp viral RNA mini kit (QIAGEN, Venlo, The Netherlands) according to manufacturer’s instructions. Isolated RNA was reverse-transcribed to cDNA with high-capacity cDNA reverse-transcription kit (Applied Biosystems, Foster City, CA, USA) according to manufacturer’s instructions. SVV cDNA covering the whole genome was amplified using Platinum Taq Polymerase (Invitrogen, Waltham, MA, USA)) and specific primers that amplify overlapping sequences of approximately 1 to 2 kb (Fwd1: TTTGAAATGGGGGGCTGG; Rev1: TTTGTGAGGAGACCCGCTAATCC; Fwd2: TTCAGTAGACTTCTCGACTCCTC; Rev2: GGGAACGAGCACCATAACG; Fwd3: GTCCCAATTTCATCAACCCCTATCAAG; Rev3: TTGTGCAGGCTAAACCAACCATCAG; Fwd4: CTACATCTCGCCCAGTGACTACC; Rev4: ACAGGCCCGAGCTTCTTCATCT; Fwd5: AAAGAGAAAGCCAGCCCTG; Rev5: TACTTCATCCAAGTCAACATCC; Fwd6: GCCGGGACCTATATCTC; and Rev6: TTTTTCCCTTTTCTGTTCCGACTG). PCR products were stored at −20 °C until further processing. Libraries for next-generation sequencing (NGS) were prepared using Nextera XT DNA Library Prep Kit (Illumina, San Diego, CA, USA) according to the manufacturer’s instructions. Library concentrations were determined using a Qubit 4 fluorometer (Thermo Fisher Scientific, Waltham, MA, USA), then diluted to a final concentration of 4 nM, pooled, and run on a MiSeq (Illumina, San Diego, CA, USA) instrument using a MiSeq v2 (300-cycle) reagent kit (Illumina, San Diego, CA, USA).

*Data processing and analysis*. Raw paired-end reads were trimmed and filtered using BBDuck [38] and aligned to the SVV reference genome with Bowtie2 [39]. Samtools [40] were used for file conversions, discarding unmapped reads, and generating BAM indexed files. LoFreq [41] was used to identify the single nucleotide variants (SNV) and the standalone program QuRe [39] was used for quasispecies reconstruction. Data analysis and visualization was performed in R v4.0.1 [42] using the R packages circlize v0.4.13 [43] and ggplot2 v2_3.3.5 [44].

SNVs were identified using SnapGene Viewer using the SVV-001 sequence NC_011349.1 as reference. Structural comparisons were made using PBD 3CJI as reference. Protein structures were visualised with UCSF Chimera and Chimera-X tools. The numeration of amino acid residues started from position one for each individual capsid protein. For comparison with other SVV isolates, EMBOSS Needle Pairwise Sequence Alignment or EMBL-EBI multiple sequence alignment tools were used.

## 3. Results and Discussion

The SVV is known to naturally infect a range of solid tumor cell lines with variable degrees of cytotoxicity [23]. Previous studies have evaluated the virus infectivity, anti-cancer efficacy, pathogenicity, and tolerability [23,45,46]. However, to date, there are no studies that evaluated the molecular changes responsible for the capacity of the SVV to adapt to a host environment by multiple passaging. 

**Figure 1:**
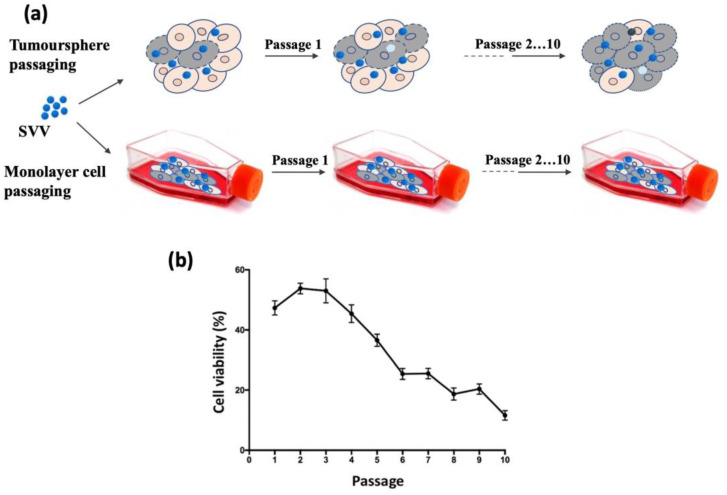
Cell viability of H446wt tumorspheres infected with SVV. (**a**) Schematic representation of the experiment. (**b**) Tumourspheres were infected for 48 h at MOI 1 with virus collected from the previous passage.

Here, we used deep genome sequencing to investigate the genetic evolution of the SVV in the tumorspheres of H446wt small-cell lung cancer cells compared to the monolayer culture over ten passages (Figure 1a). Importantly, the experiments were performed with a constant number of infectious virus particles, determined by a plaque formation assay. The repeated passaging of the SVV at a constant MOI in the tumorspheres caused an increase in the capacity of the virus to cause cell death over time, perhaps due to the virus population adaption to the new environment (fitness gain) (Figure 1b). Studies with other picornaviruses have shown that mutants that gained dominance as a result of a change in environmental constraints can be outcompeted by other sets of genomes in the same population; however, once positively selected, these viruses do not return to the original population, thus forming a sustained memory within the viral quasispecies [47,48]. The mathematic modeling of viral quasispecies indicate that it is not a collection of random misaligned genomes but rather a collection of interacting variants which build the characteristics of a viral population [49,50].

**Figure 2:**
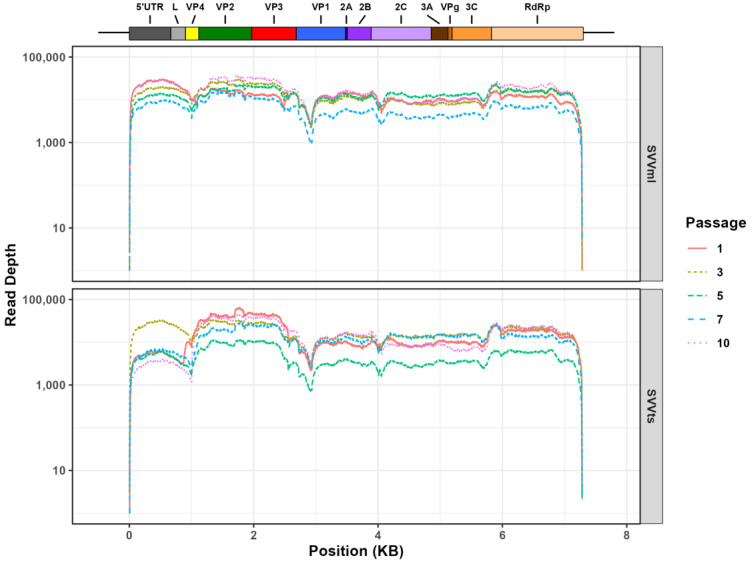
Read-depth values across the Seneca Valley virus (SVV) genome for virus populations propagated in monolayer cells (SVVml—**top** panel) and tumorspheres (SVVts—**bottom** panel) at Passage: 1 (solid red), 3 (dashed gold), 5 (double-dashed green), 7 (long dash blue), and 10 (dotted purple). SVV genome regions are indicated at the top of the figure.

Our initial data validation and filtering was conductive for the in-depth interrogation of the genetic variance between the two populations. The average read depth at each genome position across all samples was between 12.7 k and 18.3 k except for Tumorsphere Passage 5 where the average depth per read was 4.8 k (Figure 2). All samples showed a 99.4% to 99.6% genome coverage compared to the SVV reference genome.

**Figure 3:**
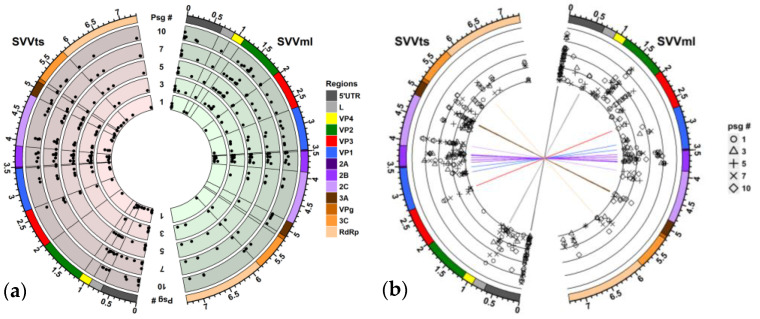
Distribution of single nucleotide variants (SNVs) in Seneca Valley virus (SVV) populations passaged in tumorspheres (SVVts) and monolayer cells (SVVml). (**a**) Circos plot of SNV distribution by passage. Point placements indicate the SNV frequency (low to high frequency from inner to outer circle). (**b**) Circos plot of SNV distribution by frequency. Concentric lines (from inner to outer circle) represent SNV frequencies of 1.0%, 3.2%, 10.0%, 31.7%, and 100% at Passage 1 (circle), Passage 3 (triangle), Passage 5 (cross), Passage 7 (diagonal cross), and Passage 10 (diamond). The outer track indicates the corresponding region on the SVV genome. Connecting lines in the center part of the plot indicate the SNVs common to both populations and are coloured based on their location on the SVV genome region. Graphic shows SNVs with >1% frequency.

The reference genome for this study was obtained from a population of the SVV propagated in H446wt monolayer cells. Overall, in the SVV population passaged in the monolayer cell culture (SVVml), the quasispecies expanded over time resulting in a larger number of single nucleotide variations (SNVs) by the tenth passage (60 SNVs in Passage 10) compared to tumorsphere-passaged SVV (SVVts) (43 SNVs in passage ten). By comparison, the first tumorsphere-passaged SVVts population appears to contain more SNVs (54 SNVs versus 30 SNVs in the first passage of SVVml) with most nucleotide changes being synonymous (Figure 3a). Our data evaluation suggests that the SVV quasispecies are evolving under experimental conditions both in the monolayer and in the tumorspheres but in different directions, with only a small number of common substitutions. These rare similarities are mainly in the non-structural viral proteins, especially the 2B genome region (Figure 2b). Having these commonalities is not surprising as both populations are derived from a common viral pool.

Conversely, the intrapopulation SNV distribution is more consistent. In both populations, most of the substitutions are recurring throughout passages with some variability of frequency (Figure 3). This finding suggests that, once a SNV is positively selected as part of the viral quasispecies, it will be propagated in the population regardless of its frequency. However, some SNVs have only a short passage occurrence suggesting a counterbalancing of negative- and positive-selection pressure factors. An analysis of the intrapopulation SNV frequency found that most mutations are under positive selection. The constant intrapopulation reoccurrence of multiple SNVs suggest that the SVV virus population, whether passaged through tumorsphere or monolayer cells, has accumulated a memory. How this memory is affecting the overall characteristics of a viral population is not fully understood, but one can speculate that it can induce a quick response if the environmental conditions are reoccurring.

**Figure 4:**
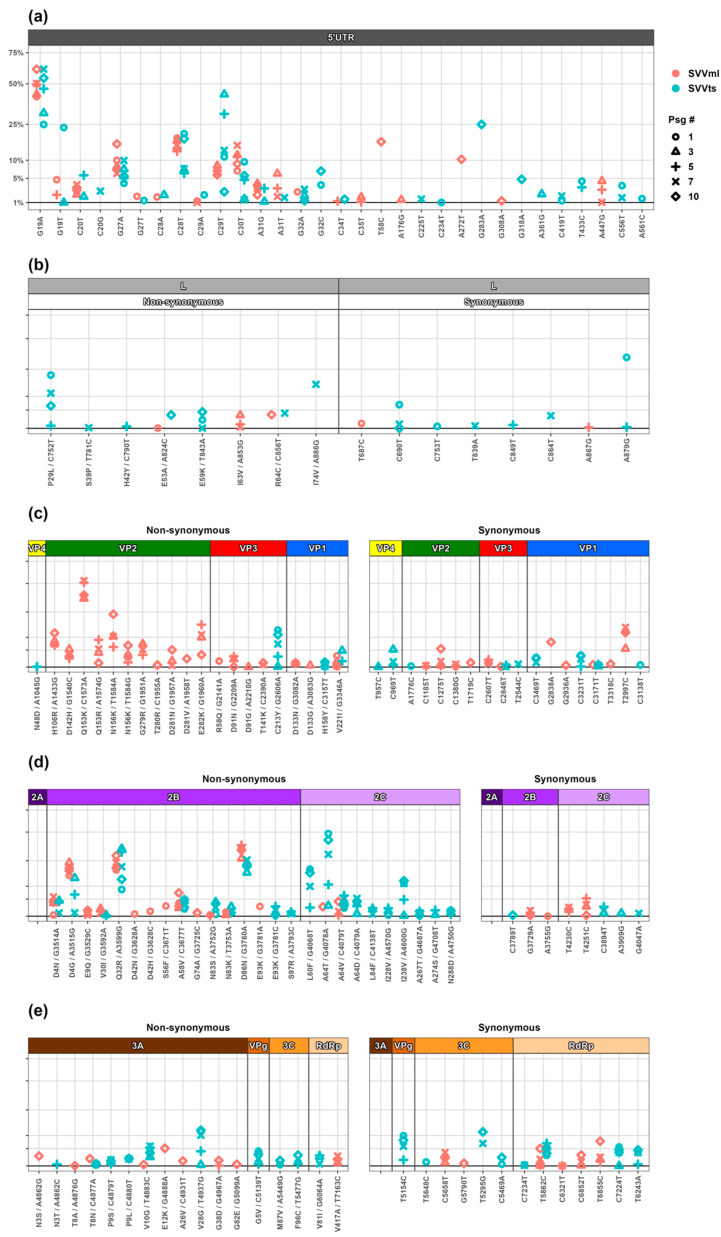
Distribution of single nucleotide variants (SNVs) in Seneca Valley virus (SVV) populations passaged in tumorspheres (SVVts) and monolayer cells (SVVml). Points represent the passages as follows: Passage 1 (circle), Passage 3 (triangle), Passage 5 (cross), Passage 7 (diagonal cross), and Passage 10 (diamond) in tumorspheres (red) and monolayer cells (blue). The x-labels denote the reference genome position and alternate allele for each SNV present at a frequency of greater than 1%. (**a**) 5′ untranslated region (UTR). (**b**) Leader protease (L) encoding genome region. (**c**) P1 genome region encoding for the structural proteins VP1, VP2, VP3, and VP4. (**d**) P2 region encoding for the non-structural proteins 2A, 2B, and 2C. (**e**) P3 region (3A, 3B-VPg, 3C-protease, and 3D-RdRp).

**The 5′ UTR region.** The SVV has a 666-nucleotide 5′-untranslated region (UTR) with a pestivirus-like internal ribosome entry site (IRES) that extends from 31 to 55 nucleotides into the coding region. The SVV IRES is not dependent on the eIF4F initiation factor, but the removal of the 31–55 nucleotide coding sequence diminishes its activity [51]. We have noticed a total of 31 synonymous SNVs in the 5′ UTR region and no non-synonymous changes. The tumorsphere-passaged SVV displayed 25 SNVs and the monolayer-passaged SVV presented a total of 20 SNVs. The nucleotides in positions 19, 20, 27, 28, 29, 31, and 32 showed multiple polymorphisms, with some of the sites recording both a positive selection resulting in increased frequency in later passages, and a negative selection and subsequent disappearance from the population (Figure 4a). For example, G19A has gained almost 50% frequency in both viral populations, while G19T appeared during early passages and quickly lost fitness and disappeared from the pool. SNVs in positions 27, 28, and 29 showed similar trends. The genome region following nucleotide position 35 showed no SNVs.

To understand the role of this conservation, we blasted the SVV 5′ UTR sequence and selected 100 hits that had more than 95% similarity. A multiple sequence alignment and evolutionary tree assembly of the 5′ UTR region grouped the SVV USA isolates into seven major clusters. Considering our reference genome closest to the USA strains, the evolutionary tree suggests a constant evolution in the 5′UTR associated with substitution. It is interesting that our SVV populations selected for the same positively selected positions, suggesting that these mutations are important for the virus. However, the manner in which these mutations favour the quasispecies within both SVV populations needs further investigation. To understand if positively or negatively selected nucleotides lead to a structural change, we created 2D/dot bracket models (ViennaRNA package 2.0) [52] and 3D models (3dRNA 2.0) [53,54] of the 5′ UTR region containing the mutations of interest in several combinations, but no structural change was observed. This suggests that the structural integrity of the 5′ UTR is important for the SVV’s survival. Predicted RNA structures of the 5′ UTR showed the first 100 nt to be involved in the formation of a double stem loop. To understand if the negatively selected mutations at the polymorphic nucleotide positions influence the stem-loop formation, we modelled the predicted structure with mutations G19T, G27T, C28A, and C29A separately and in combination. Whether examined individually or simultaneously, there was no disruption of the stem-loop formation. However, we have noticed the reduced spatial compactness of stem-loop1 localized immediately at the start of the 5′ UTR. In RNA viruses, genome packaging requires the structural compactness to fit in a small protein shell. Studies have shown that RNA viruses have evolutionary pressure towards the compactness of the RNA size [55] and the overall viral RNA compactness is directly proportional to the compactness of RNA secondary structure such as stem-loops [56]. We hypothesise that the loss of compactness at the stem-loop level is negatively favouring the selection in this case.

**Leader protease (L^pro^).** Leader protease is found only in a select group of picornaviruses that include cardioviruses, aphthoviruses, and the Seneca Valley virus. The foot-and-mouth disease virus (FMDV) L^pro^ is defined as a papain-like cysteine protease that self-cleaves from the viral polyprotein. The viral protease is responsible for the cleavage of eukaryotic translation initiation factors 4GI and 4GII and subsequently switching off the host cell cap-dependent protein synthesis [57,58]. However, the function of the SVV L^pro^ is not known. The region differs from other picornaviruses L^pro^ by lacking the catalytic residues necessary for proteolytic activity [24].

Overall, under our experimental conditions, the SVV L^pro^ remained conserved in both viral populations. The serial passaging of the SVV in the tumorspheres resulted in six synonymous and seven non-synonymous L^pro^ mutations. Among the non-synonymous mutations, five occurred only once, suggesting a negative selection of these traits, while P29L and E59K became established in the population. The monolayer-passaged SVV population showed even less variation, with only three non-sustained amino acid substitutions, suggesting a trend of negative selection (Figure 4b).

**P1 region.** The P1 region codes for the structural proteins that form the capsid structure of the SVV. There are four structural proteins in SVV—VP1, VP2, and VP3 that form the exterior of the full virion, and VP4 found on the interior of the capsid. These proteins are not only important for protecting the viral genome but are vital for receptor interactions and immune responses in the host [59,60]. Changes in the capsid proteins are linked to both the gain and loss of viral function [61,62]. We have identified only four amino acid substitutions in the capsid region in the tumorsphere-passaged SVV population, none of which were localized in the VP2 protein. By contrast, we found 18 amino acid substitutions in the monolayer-passaged SVV population, of which eleven are in VP2 (Figure 4c). Picornaviruses make frequent genome replication errors, resulting in the accumulation of mutations in viral capsids over time [63]. However, our finding that the VP2 region has not accumulated any mutation in the tumorsphere-passaged SVV population challenges the concept that mutations in picornaviruses are a result of a random error due to the RdRp low fidelity. Out of eleven VP2 amino acid substitutions in the cell monolayer-passaged viral population, nine are well-established, with Q153 and D281 showing polymorphism. It is pertinent to notice that most of the VP2 substitutions are to positively charged residues (either K or R). In contrast to the high mutational profile of the VP2, the other capsid proteins do not show any high-frequency and fully established substitutions except for V221I in VP1 that stayed at a low frequency (under 5%) and showed no gain of fitness over time. To our knowledge, there are no studies on picornavirus-directed evolution and mutation profiling in tumorspheres. However, a recent study that evaluated the evolution of the SVV in vitro in a baby hamster kidney monolayer cell culture identified three amino acid substitutions in VP2, two in VP3, and four in VP1, respectively [64]. Interestingly, H256R in VP2 was also identified in our study in the monolayer-passaged population (Figure 4c). Overall, the rate of synonymous mutations in the P1 region is similar in both populations. Most of the non-synonymous mutations occurring in the P1 region are positively selected and either gained fitness or maintained their frequency from passage to passage. A large-scale genetic and evolutionary analysis of the current and historic SVA genomes identified multiple positively selected pressure sites [65]. Interestingly, several of these positions were also modified in our study (N156, G279, and T280 in VP2; R58 and D91 in VP3; and V221 in VP1, corresponding to codon positions 306, 429, 430, 492, 515, and 894, respectively) and showed sustainability in the quasispecies population, confirming the positive selection of changes at these sites during SVV evolution.

**P2 region.** The P2 genome region of picornaviruses codes for the non-structural viral proteins 2A, 2B, and 2C. The picornavirus 2A proteins are highly variable and are classified based on their structure and function into five groups: chymotrypsin-like 2A, parechovirus-like 2A, HAV-like 2A, aphthovirus-like 2A, and cardiovirus-like 2A [66]. The 2A is linked with host translation shutdown and the inhibition of apoptosis, impacting viral survival [67,68]. The SVV 2A is a small protein of nine amino acids with its role predicted to be limited to a ribosome-skipping function [24,69]. Our analyses show no mutations in the 2A region of either tumoursphere- or monolayer-passaged SVV populations (Figure 4d), suggesting that the conservation of this region is important for virus replication. Our findings are consistent with a study that evaluated 33 virus isolates against the prototype SVV strain and reported no mutations in the 2A region [70]. Similarly, the evolution of an SVV isolate in 80 monolayer cell passages found no mutations in 2A [64]. These findings further strengthen the hypothesis that the 2A region is under high negative-selection pressure.

The 2B protein is an important viroporin and is associated with viral replication and release from host cells [71,72,73]. The 2B is also a major modulator of the cellular Ca^2+^ concentration, resulting in the modification of membrane permeability, autophagy, apoptosis, and modulation of the host immune system [74,75]. We have observed that the rate of non-synonymous mutations is significantly higher in the 2B region compared to the rate of synonymous mutations (Figure 4d). There are 10 AA substitutions in the SVVts population and 14 AA substitutions in the SVVml population. Almost all SVVts AA substitutions in the 2B region appear to be repeated between passages, suggesting either positive selection or adaptive mutations. In contrast, in the 2B protein of the SVVml population, half of the mutations are negatively selected and disappear from circulation within one passage. Fully established, presumably positively selected AA substitutions are present in both populations at similar frequencies (D4N, D4G, Q32R, A58V, and D86N). Notably, the N83 and E93 substitutions in the 2B region are also reported in other evolutionary studies of the SVV from pig isolates [64], suggesting that these two mutations have a broader distribution in SVV global genomes and are established in the wild-type populations. A poliovirus (PV) study that evaluated virus adaptation to cell culture identified the 2B region as a hot spot for genetic variation and suggested that mutations are adaptive and specific to the type of tissue/cells in which the virus is propagated [76]. In relation to the SNV sites and codon preference, a study of FMDV synonymous codon usage suggested that mutation pressure is vital for the preference of codons used in the 2B genome area [77]. SVV 2B is shown to suppress type-I interferon, suggesting an SVV 2B role in host immunity modulation [78]. The 2B mutations gaining fitness in the tumorsphere-passaged SVV could be responsible for an increased efficacy in manipulating the host immune defences as indicated by the increased cytotoxicity in the later passages. However, the role of these mutations cannot be predicted based on mutation analysis alone. It can be inferred from the available evidence that 2B is a hot spot for non-synonymous mutations for the SVV and some of the identified substitutions are established in other SVV isolates [64]. The gain of unique substitutions in the 2B region indicates that this region could be a determinant of adaptation to various host models.

Along with 2B, SVV 2C is also shown to modulate the host cell innate regulators. The SVV 2C has been shown to regulate the RIG-I-induced IFN-ß [79]. We noticed that the SVVts population showed a significantly higher number of non-synonymous mutations compared with the SVVml population. There are 10 AA substitutions in the SVVts population, with one polymorphic site (A64) in the 2C region, which shows a significant gain of fitness over time, suggesting a favoured selection. The remainder of the identified mutations in the SVVts population are at relatively stable frequencies, suggesting they are also favoured at low frequencies. In contrast, there are only two non-synonymous mutations in the SVVml population, and these appear to be negatively favoured. We have compared this region with previously reported SVV evolutionary studies and found no similarities. Furthermore, we have observed that the rate of non-synonymous mutations in the SVVts population is higher than previously reported or compared with the SVVml passages. This might be attributed to the tumorsphere environment where different constraints are applied to viral evolution compared to growth in single-cell monolayers. Once a virus is in the tumor environment, it is somewhat protected from host antibodies as antibody internalization and clearance is prevented by solid tumors and limited vascular permeability [80]. However, for successful anti-tumor activity, an oncolytic virus needs to overcome cellular defense mechanisms. It has been shown that SVV 2C inhibits the innate host immune response by the downregulation of type-I interferon production via mitochondrial antiviral-signaling protein degradations [81]. The evidence that the SVVts population shows improved antitumor activity indicates that positively favoured 2C substitutions could be a potential contributor to the enhanced SVV antitumor activity seen in our study. In future studies, the identified substitutions should be tested in different models to validate the mechanism of enhanced cell killing.

**P3 region.** The P3 genome region of picornaviruses is essential for viral replication. P3 codes for four non-structural proteins (3A, 3B, 3C, and 3D). The 3A is a highly variable protein in the picornavirus family and forms the N-terminal of the P3 precursor [82,83]. Both 3A and 3AB were shown to have multiple roles during viral replication [83]. In coxsackievirus B3, the 3A protein is associated with the inhibition of endoplasmic reticulum (ER)-to-Golgi transport [84]. The mutagenesis of the 3A protein in picornaviruses is linked with a hampered RNA replication and specific amino acid substitutions are linked with changes in host tropism and range [84,85,86,87,88,89]. The role of SVV 3A is not yet established. However, in our study, we identified six AA substitution positions in the SVVts population with V28G gaining fitness in the population (Figure 4e). At the same time, seven out of eight AA substitutions in the SVVml populations have emerged only in Passage 10 and it is not possible to predict if these mutations would have been sustained and under positive or negative selection in subsequent passages. Although we have identified a total of 12 AA substitution positions in both populations, their role remains to be established as there are multiple structural and functional variations between the 3A proteins of picornaviruses [83]. The 3B of SVV is a small genome-linked protein (VPg) and serves as a primer for viral RNA synthesis. The SVV VPg has low homology with other members of its family [24,69]. We have noticed only one AA sustained substitution in the SVVts population, and no mutations in the SVVml population. To our knowledge, there are no reports to date on the effects of SVV VPg mutations, but other picornavirus members have been shown to have reduced infectivity and changes in cytopathology [90].

To complete the replication and infection cycle, viral proteases 3C and 3CD are instrumental in the release of mature and functional viral proteins. The 3C protease (3Cpro) is linked to the initiation of RNA synthesis, the impediment of host protein synthesis, the hindering of the nucleus–cytoplasmic transport, and cell apoptosis [91,92,93,94]. We have observed only two non-synonymous mutations in the SVVts populations and none in the SVVml populations. The viral RNA-dependent RNA polymerase (RdRp) 3D is vital for the viral transcription and replication, and is also responsible for the VPg uridylation, a process that initiates viral replication [95,96,97,98]. We have observed only two AA substitutions in the 3D region: V417—which is sustained in the SVVml population, and V81—which seems to lose fitness over time, in the SVVts population. The mutation frequencies that we have noticed for RdRp are at a low-frequency level. Moreover, the mutation detected in the SVVts showed a negative-selection trend, suggesting a random AA substitution. The contribution of the sustained RdRp mutation in the SVVml population to the dynamics of the viral swarm cannot be predicted with the mutational analysis only. The mutational analysis of FMDV and PV have demonstrated that substitutions at the N-terminus of RdRp, away from the active site, are linked to the disruption of the natural fidelity of the polymerase [99,100,101,102].

## 4. Conclusions

In summary, we passaged the oncolytic virus SVV in the small-cell lung cancer cell line H446wt using two cell culture models: standard 2D cell monolayers and tumorspheres. Deep genome sequencing analysis of virus populations collected every second passage for ten passages identified a total of 150 SNVs across the genome, with 72 AA substitutions, of which 12 substitutions showed polymorphism. We observed that the SVVts population showed a high degree of conservation across the P1 region, especially at VP2 where not a single non-synonymous mutation was observed. In comparison, the SVVml population showed increased diversity in this region overtime. In contrast, the P2 region showed a greater mutation rate for the SVVts population compared to SVVml. In the P3 region, the overall mutation rate is relatively similar in both virus populations. However, the SVVml presented several mutations in the last passage only that contributed to matching the mutational score with the SVVts population, which has showed multiple sustained mutations in this region. Two striking features of the SVVts population are: (i) VP2 is completely conserved, and (ii) there is a high mutational rate in the P2 region, especially for the 2C protein. This might suggest that the increase in the ability of the SVV to kill cells over time in the tumorspheres is acquired by capsid conservation and positively selecting mutations to counter the host innate immune responses in the tumor environment. The only fully conserved protein is 2A in both populations.

We have demonstrated that the serial passaging of the SVV in a tumor-mimicking microenvironment can quickly modify the original viral quasispecies population, which could naturally enhance the anti-tumor activity of the virus over time.

## Data Availability

The data presented in this study are available on request from the corresponding author.
